# Advanced vs. Standard Monofocal IOLs: Optical Quality and Patient-Perceived Visual Outcomes

**DOI:** 10.3390/jcm14176255

**Published:** 2025-09-04

**Authors:** Carla Charbel, Lidia Pérez-Sanz, Nuria Garzón, Francisco Poyales, Jesús Carballo

**Affiliations:** 1 Optometry and Vision Department, Faculty of Optics and Optometry, Complutense University of Madrid, C/Arcos de Jalón, 118, 28037 Madrid, Spain; ccharbel@ucm.es (C.C.); lidiampe@ucm.es (L.P.-S.); jcarball@ucm.es (J.C.); 2 Miranza IOA Clinic, C/Galileo, 104, 28003 Madrid, Spain; p.poyales@gmail.com

**Keywords:** advanced monofocal, cataract, ISOPure, optical quality, quality of vision

## Abstract

**Background/Objectives:** The objective of this study is to compare the optical and visual quality provided by the advanced monofocal intraocular lens (IOL) ISOPure and the standard monofocal IOL MicroPure in cataract patients, using objective and subjective assessments. **Methods:** This prospective, single-blind clinical study includes 28 patients with cataracts, bilaterally implanted with either the ISOPure or MicroPure IOL. Eligible eyes had no ocular comorbidities and regular corneal astigmatism ≤ 1.00 D. Three months postoperatively, uncorrected distance and intermediate (UDVA, UIVA) and corrected distance and intermediate (CDVA, DCIVA) visual acuities were measured at 4 m, 80 cm, and 66 cm under photopic (85 cd/m^2^) and mesopic (3.5 cd/m^2^) conditions. Photic phenomena, including halo and glare, were evaluated. Objective optical quality was assessed using Objective Scattering Index (OSI), Modulation Transfer Function (MTF), Strehl Ratio (SR), and ocular aberrations. Subjective patient satisfaction was evaluated using Quality of Vision (QoV) and Catquest-9SF questionnaires. **Results:** Under photopic conditions, logMAR DCIVA at 80 cm, UIVA at 66 cm, and DCIVA at 66 cm were 0.18 ± 0.06, 0.25 ± 0.12, and 0.20 ± 0.13, respectively, for ISOPure, and 0.22 ± 0.06, 0.30 ± 0.09, and 0.25 ± 0.09 for MicroPure (*p* = 0.05, 0.02, and 0.05, respectively). No significant differences were observed in halo/glare size or intensity, OSI, MTF, or SR. However, statistically significant differences were found in higher-order total aberrations for pupil sizes of 3.0, 4.0 mm, and 5.0 mm. Questionnaires indicated greater satisfaction and functional intermediate vision with ISOPure. **Conclusions:** The ISOPure IOL offers superior intermediate vision without compromising distance vision, delivering a balanced combination of optical quality, functional performance, and patient satisfaction.

## 1. Introduction

Cataract is the most common cause of reversible vision loss; however, normal visual function can be restored to date by only using surgery with the use of an intraocular lens (IOL) [1]. Most IOLs implanted worldwide are monofocal [2]. However, intermediate vision has become increasingly important due to the widespread use of electronic devices, such as computers, tablets, and mobile phones, which require sustained visual focus at that distance. In this context, improving intermediate vision has become a priority objective in both presbyopia correction and cataract surgery.

To improve intermediate vision, recent advances in intraocular lens technology have led to the development of enhanced monofocal designs. These lenses aim to improve performance at intermediate distances while maintaining quality distance vision. However, most patients implanted with these lenses still require spectacles for near tasks. This type of lens typically features optics that extend the depth of focus in a way similar to extended depth of focus (EDoF) lenses, although to a lesser extent. Currently, there is no clear regulation that objectively distinguishes between these types of lenses.

Even though many manufacturers label their lenses as EDoF, the definition of EDoF has been established by the American National Standard Z80.35–2018 (ANSI) [3], which specifies four effectiveness endpoints that must all be met in order for an IOL to be classified as EDoF. Advanced monofocal lenses do not meet these ANSI criteria, and are therefore generally categorized as enhanced monofocal lenses.

One such lens classified as enhanced monofocal is the ISOPure IOL (BVI medical, Liége, Belgium). Its design is based on the precise modification of spherical aberration through controlled alterations in the geometry of the optical surfaces. According to the designers, the lens incorporates isofocal technology, which combines monofocal refractive optics with a complex polynomial surface profile. It uses higher-order conic surfaces and a refined balance of spherical aberrations that are precisely adjusted for each diopter across the optic [4].

Several publications have analyzed this lens, although results are often contradictory when comparing optical bench data with clinical outcomes. Optical bench studies suggest that, due to its design, the lens may produce larger halos than other types of lenses, as reported by Labuz et al. [5], or show worse MTF values under larger pupil sizes, as found by Alarcon et al. [6]. However, other authors, such as Ang et al. [7], have shown that patients report good clinical outcomes, even when assessing halos or under mesopic conditions, as reported by Pérez-Sanz et al. [8].

In this context, the present study aims to evaluate both the optical quality achieved after implantation and the visual quality perceived by patients, using objective and subjective measurements and satisfaction surveys, in patients implanted with the advanced monofocal IOL ISOPure compared to those implanted with the standard monofocal IOL MicroPure (BVI medical, Liége, Belgium). Both lenses are made from the same hydrophobic biomaterial and differ only in optic design.

## 2. Materials and Methods

This prospective clinical study adhered to the tenets of the declaration of Helsinki. The study protocol was approved by the San Carlos Clinical Hospital review board (code: 20/030-R_P) and written informed consent was obtained from all patients. This study was designed as a single-blind study, where patients were not informed whether they were implanted with the study lens or the control lens, but only that they would receive either a monofocal or an advanced monofocal IOL. The ophthalmologist knew the implanted lens during the surgery, while the optometrist was unaware of the result of the randomization.

The sample size was calculated based on the previous study performed by Ruiz-Mesa et al. [9]. The article evaluated an EDoF lens using the same halometer as in the current study. An alpha risk of 0.05 and a beta risk of 0.2 were accepted. Thus, it was determined that 14 subjects would be required in each group to detect a statistically significant difference equal to or greater than 0.06 units in the discrimination index.

Twenty-eight patients undergoing bilateral cataract surgery at Miranza IOA Clinic (Madrid, Spain) were recruited. They were randomized equally to either study group bilaterally implanted with the ISOPure lens under investigation, or with the control lens (MicroPure). The randomization was given by a software system. Inclusion criteria were: age 50 years or older, cataractous eyes with no comorbidity, a regular corneal astigmatism ≤ 1.00 D and calculated IOL power within the range of the analyzed IOLs, preloaded from 10.00 D to 30.00 D in steps of 0.50 D.

All surgeries were carried out by the same ophthalmologist (FP) under topical anesthesia. Anterior capsulotomy and nuclear fragmentation were performed with a femtosecond laser (CATALYS Precision System, Johnson & Johnson, Santa Ana, CA, USA). A 2.2 mm corneal incision and a paracentesis were made with a surgical knife, while for lens phacoemulsification a commercial microsurgical system (Centurion Vision System; Alcon Laboratories, Inc., Fort Worth, TX, USA) was employed. The IOL’s power was calculated by applying the Barrett Universal II formula for emmetropia.

### 2.1. Intraocular Lens

The advanced monofocal IOL ISOPure was implanted in the study group, while the standard monofocal MicroPure was the IOL implanted in the control group. ISOPure is 100% refractive and is characterized by its optic design based on the patented isofocal concept [10]. This concept refers to an optic with smooth anterior and posterior surfaces steepening in the center and smoothly varying alternating power, so that they create a practically constant high image quality in a range of focus while being relatively pupil independent. These optical features help to extend the depth of focus compared to monofocal IOLs, while maintaining a good far focus performance. According to some publications, on the optical bench, ISOPure tends to achieve around 1.00 D of EDoF, 50% superior to standard aspheric monofocal IOL [11].

Both IOLs are based on identical four closed haptics platform and made of acrylic chemically crosslinked polymer biomaterials with incorporated UV and blue light filters. IOLs made of this hydrophobic biomaterial (GFY, technical name) are recognized for their capsular biocompatibility due to material tackiness and bio-adhesiveness, which help prevent posterior capsular opacification. Furthermore, GFY shows a high refractive index (1.53), allowing for the manufacturing of thinner lenses implantable through a small incision size, thus rapid eye restoration [12].

### 2.2. Protocol Evaluation

Although both eyes were operated on, only one eye was included in the clinical assessments. Only the questionnaire responses were based on binocular vision. LogMAR uncorrected and corrected visual acuities for distance at 4 m (UDVA and CDVA), as well as for intermediate distances (UIVA and CIVA) at 80 and 66 cm, were obtained under photopic (85 cd/m^2^) and mesopic (3.5 cd/m^2^) luminance conditions.

Halo size was measured using the open access software package Halo V1.0 (Laboratory of vision sciences and Applications, University of Granada, Spain). As already shown in previous papers, this psychophysical test allows to study the effect of the experimental conditions in the discrimination capacity of peripheral stimuli in the presence of visual disturbances of the subject quantifying the influence of halos perceived by the observer [9,13]. It is evaluated by a parameter called discrimination, ranges from 0 to 1, where 0 indicates high effect of halos and 1 indicates no effect. So that the higher the discrimination index, the less the effect of halos. The test placed at 4 m from the patient, was presented under scotopic conditions, during which the patient identified peripheral light stimuli that emerged randomly around a central spotlight.

A halo and glare software simulator (Eye Design Network GmbH, Vreden, Germany) was used to assess the subjective perception of photic phenomena. The patients were asked to adjust the photic phenomena level in a simulated image to the amount they perceive such photic phenomena, giving a representation of how they perceive halos around light sources during nighttime driving. The slide bar position for each item is translated into numeric values between 0 (minimum) and 100 (maximum) on a visual analog scale.

Optical quality parameters were also evaluated, including the Objective Scattering Index (OSI), the Modulation Transfer Function (MTF), and the Strehl Ratio (SR), using the HD Analyzer (Keeler, CA, USA), which is based on the double-pass technique. Regarding aberrations, total higher-order aberrations were measured using the OPD-Scan III aberrometer (Nidek Technologies, Gamagori, Japan) at 3.0, 4.0 and 5.0 mm pupil diameters.

Contrast sensitivity defocus curves were assessed using the Multifocal Lens Analyzer (QVision, Almería, Spain) [14]. For this test, patients’ pupils were dilated with tropicamide, and calibrated aperture diaphragms mounted in a trial frame 10 mm from the corneal vertex were used to simulate the entrance pupil. Two contrast sensitivity defocus curves were measured with pupil diameters of 3.0 mm and 4.5 mm, respectively, under photopic conditions, with the optotype displayed at a distance of 2 m. Trial lenses ranging from −2.50 D to +1.00 D in 0.50 D increments were placed. To shift the defocus point from infinity to a testing distance of 2 m, a +0.50 D spherical lens was inserted.

Finally, to assess patient satisfaction after IOL implantation, two questionnaires were administered: The Quality of Vision (QoV), focusing on the perception of photopic phenomena, and the Catquest-9SF questionnaire, centered on difficulties the patient may experience in daily visual tasks.

### 2.3. Statistical Analysis

All statistical tests were performed using the SPSS software package for Windows (version 22.0, SPSS Inc., Chicago, IL, USA). First, basic descriptive statistics were calculated for quantitative variables, including the mean, standard error, and maximum and minimum values, in order to provide an overview of the data. The Shapiro–Wilk test was used to assess data distribution and determine whether it followed a normal distribution.

When the data met the assumptions of normality, parametric tests, such as Student’s *t*-test, were used to compare means between the study group and the control group. Conversely, when the data did not follow a normal distribution, non-parametric tests, such as the Mann–Whitney test, were applied to assess differences between the two groups. To evaluate associations between qualitative variables, the chi-square test was used, and the corresponding *p*-value was determined. Differences were considered statistically significant when the *p*-value was less than 0.05.

## 3. Results

A total of 28 patients were recruited and divided into two groups: the study group (ISOPure) and the control group (MicroPure). No statistically significant differences were found when evaluating demographic and preoperative ocular data, as shown in Table 1.

Visual acuity values obtained at different distances under photopic and mesopic luminance conditions are shown in Table 2. In all measurements at both 80 and 66 cm, under both photopic and mesopic conditions, the best results were obtained with the ISOPure IOL. However, statistically significant differences were found only when evaluating UIVA at 66 cm between the two lenses. The postoperative spherical equivalent was −0.03 ± 0.27 D (range −0.37 to 0.75) for the ISOPure group and 0.07 ± 0.20 D (range 0.08 to 0.50) for the MicroPure group, with no statistically significant differences found (*p* = 0.11).

Table 3 presents the postoperative measurements obtained using the Halo V1.0 program and the halo and glare simulator. No statistically significant differences were found in any of the parameters related to the size or intensity of halos or glare.

Table 4 shows the visual quality values and total higher-order aberrations (HOA) for different pupil diameters. Regarding the visual quality parameters (OSI, MTF, and SR), no statistically significant differences were found. However, when analyzing HOA, statistically significant differences were observed between the two lenses, for pupil sizes of 3.0, 4.0, and 5.0 mm, respectively.

**Figure 1:**
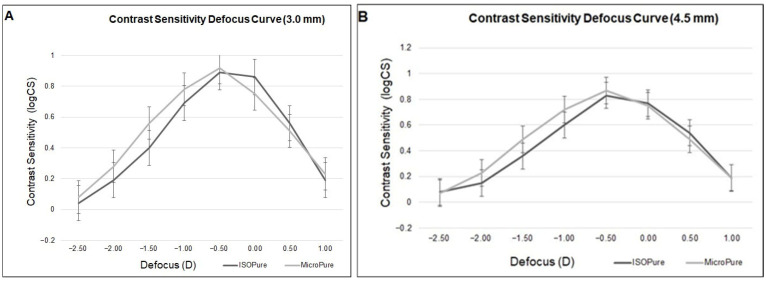
Contrast sensitivity defocus curves for the two intraocular lenses with a 3.0 mm pupil (**A**) and a 4.5 mm pupil diameter (**B**).

Figure 1 shows the contrast sensitivity defocus curves for the two lenses with a 3.0 mm pupil (Figure 1A) and a 4.5 mm pupil (Figure 1B). No statistically significant differences were found between the two lenses for any of the vergences analyzed.

**Figure 2:**
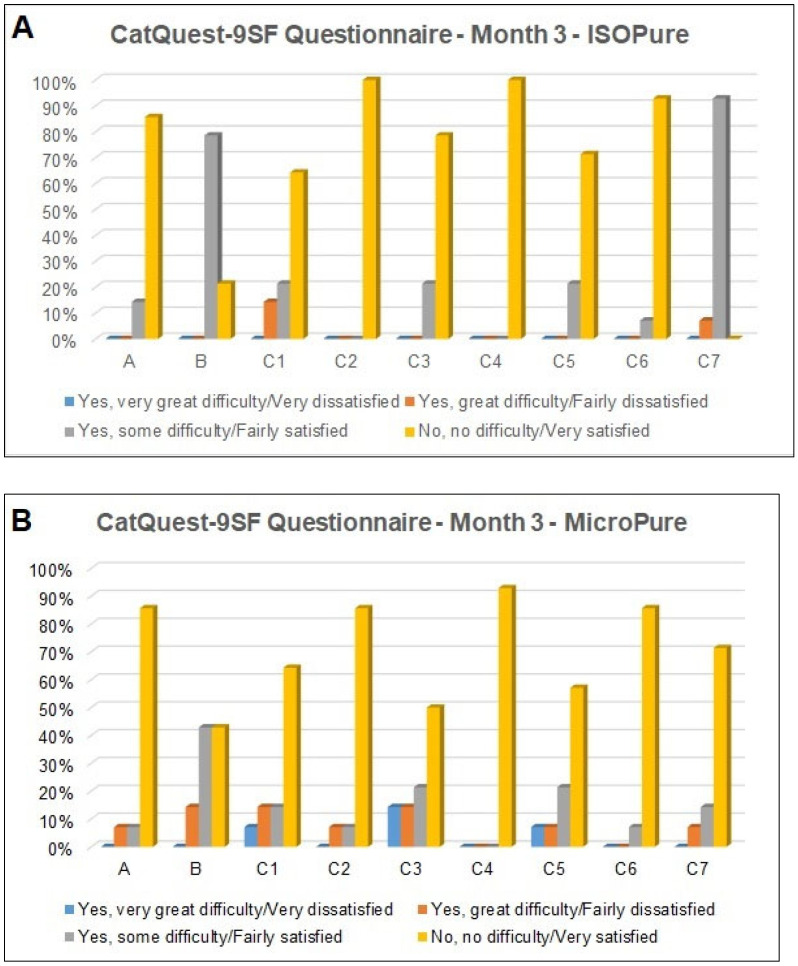
Results of the CatQuest-9SF questionnaire at the Month-3 postoperative visit for ISOPure (**A**) and MicroPure IOLs (**B**).

Finally, Figure 2 and Table 5 present the results obtained after patients completed the different questionnaires.

Figure 2 shows the CatQuest results. At three months, the responses indicated a high level of visual function in both groups (Figure 2). In the ISOPure group, all participants were able to perform daily activities without major difficulties, and all expressed satisfaction with their vision, reporting good visual function in everyday life. In addition, more than 60% reported no difficulty reading newspapers, and more than 80% reported no difficulty reading TV subtitles (Figure 2A).

In the MicroPure group, most participants also reported good visual function in everyday life, with 85.7% expressing satisfaction with their vision and high percentages reporting no difficulty across the evaluated activities (Figure 2B). Overall, both lenses allowed patients to carry out daily visual tasks effectively, with no statistically significant differences between groups.

A: Do you find that your sight at present causes you difficulty in your everyday life?
B: Are you satisfied or dissatisfied with your sight at present?
Do you have difficulties with the following activities because of your sight?
C1: Reading text in newspapers.
C2: Recognizing faces of people you meet.
C3: Seeing the process of goods when shopping.
C4: Seeing to walk on uneven surfaces.
C5: Seeing to do handicrafts, woodwork.
C6: Reading subtitles on TV.
C7: Seeing to engage in an activity/hobby that you are interested in.

Table 5 shows the outcomes of the Quality of Vision (QoV) test at 3-month post-implantation. Patients in both groups did not report notable complaints of glare, halos, double vision, or distorted vision. Regarding the frequency, intensity, and degree of discomfort when perceiving starburst, more than 70% of patients in the ISOPure group reported never experiencing it, in contrast to the MicroPure group, where 20% never reported it and more than 60% reported perceiving glare occasionally, with mild frequency and discomfort levels. In this regard, statistically significant differences were found.

## 4. Discussion

Improving intermediate vision has become a priority objective both in presbyopia correction and in cataract surgery. Thus, the success of these procedures is no longer measured solely by the restoration of distance vision, but also by the ability to efficiently and comfortably perform daily activities that require optimal optical performance at intermediate distances. According to a recent study that carried out an analysis of daily visual habits in a presbyopic population, the average time dedicated to intermediate vision was 30.2 percent, although in some patients it reached up to 50 percent [15]. These results were consistent with a previous study, which showed a mean working distance of 82.5 cm and a mean mobile phone usage distance of 31.9 cm in a sample of 454 participants [16].

In the present study, a comprehensive evaluation was conducted encompassing not only visual acuity measurements but also the optical and visual quality provided by two intraocular lenses of different designs: an isofocal optic lens, the ISOPure, classified as an advanced monofocal, and a conventional monofocal lens, the MicroPure. Both lenses share the same material and platform, so any differences observed can be attributed exclusively to the optical design. This comparative analysis allowed us to assess not only visual performance at various distances, but also objective and subjective aspects related to visual perception, optical quality, and postoperative satisfaction.

In this study, VA was assessed under different lighting conditions (photopic and mesopic). The results show that, under photopic conditions, UDVA and CDVA were similar in both groups, with no statistically significant differences. This suggests that implantation of the ISOPure lens does not compromise distance vision compared to a conventional monofocal lens. These findings are consistent with previous studies by Stodulka et al. [17], Ang et al. [7], and Bova et al. [18], which also reported similar distance VA values in isofocal and monofocal IOLs.

On the other hand, monocular intermediate vision yielded better results with the ISOPure, although differences reached statistical significance only at 66 cm. These results are in line with those reported by Mencucci et al. [19], who also highlighted the ability of ISOPure to provide stable and sustained improvement in intermediate vision over time. The findings of the present study are comparable to those of Bova et al. [18], who recorded a monocular UIVA of 0.24 ± 0.11 logMAR at 66 cm under photopic conditions.

The evaluation of mesopic VA is clinically relevant, as it reflects the patient’s ability to adapt to different light levels, an essential factor for visual performance in activities such as night driving or using electronic screens in dimly lit environments. Under these conditions, monocular distance VA remained comparable between groups, with no significant differences. However, for intermediate vision (UIVA at 66 cm), a slight superiority was observed with the ISOPure, although without clear clinical relevance. Pérez-Sanz et al. [8] reported similar monocular intermediate VA values for the ISOPure group, under mesopic conditions, at testing distances of 80 and 66 cm, consistent with the results obtained in the present study.

Regarding the evaluation of halos, both through direct observation and when using a simulator, no statistically significant differences were recorded between the ISOPure and MicroPure groups in terms of halo intensity, glare size, or glare intensity. In the study conducted by Ang et al. [7], the mean values for halo and glare size and intensity in the ISOPure group were similar to those reported for the same lens in the present study. Likewise, no statistically significant differences were observed between the ISOPure and MicroPure groups for these parameters. This finding, together with the consistency of results between our study and that of Ang et al. [7], suggests that the subjective perception of dysphotopsia phenomena, such as halos and glare, remained similar in both groups, without differences in clinical impact. The low incidence of clinically relevant halos and glare suggests good tolerance to the optical design of both IOLs, which could contribute to a more uniform, comfortable, and predictable visual experience, particularly under low-light conditions such as those encountered in night driving.

When considering patient satisfaction with dysphotopsia phenomena, as reflected in the Quality of Vision questionnaire, participants reported a high degree of satisfaction, without indicating that halos or glare had a significant impact on them. The ISOPure group showed better results regarding the reduction in starburst. Remarkably, this indicates that the clinical findings correspond with patient-reported outcomes, suggesting that halos observed in optical bench studies, such as those reported by Alarcon [6] or Labuz [5], are not perceived by patients in a way that causes discomfort.

In the present study, no statistically significant differences were found between the ISOPure and MicroPure groups in objective optical quality parameters, including OSI, MTF, and SR. Pérez-Sanz et al. [20]. reported MTF and SR values very similar to those obtained here. In contrast, Alarcon et al. [6], in an optical bench study, reported that ISOPure lenses showed a reduction in MTF values with larger pupil diameters, suggesting a loss of optical quality under mesopic conditions, possibly due to increased optical aberrations in the peripheral zones of the lens design. However, in the present study, no significant reduction in MTF was observed under mesopic conditions, nor were differences found between the two groups, suggesting that the effects seen in experimental settings do not manifest in a clinically relevant way in practice.

The discrepancies between clinical and optical bench studies may be explained by differences in measurement environments: while the optical bench simulates external conditions in an idealized and controlled system, clinical measurements reflect optical performance in the real physiological context of the human eye, where factors such as pupil dynamics, the Stiles–Crawford effect, and neuroadaptation, which have been previously described, may modulate the impact of aberrations induced by the ISOPure’s optical design.

When evaluating HOAs, statistically significantly higher values were found in the ISOPure group compared to the MicroPure group, particularly for pupil diameters of 4.0 mm and 5.0 mm. These results are consistent with those reported by Pérez-Sanz et al. [8], also in an optical bench setting, where Zernike coefficients showed progressively more negative spherical aberration values, both primary and higher-order, as the analyzed optical diameter increased, especially for 4.0 and 5.0 mm pupils, similar to our findings. This increase in aberrations is precisely the optical principle by which the ISOPure lens extends its depth of focus to improve intermediate vision compared to a standard monofocal model.

In the present study, the contrast sensitivity curve obtained with a 3.0 mm pupil diameter showed a typical profile, with peak values near emmetropia and a progressive decline at the ends of the defocus curve, particularly at −2.50 D and +1.00 D, in both groups. Similarly, the CS curve with a 4.5 mm pupil diameter revealed a comparable pattern. Although the MicroPure lens tended to show slightly higher CS values, with both pupil sizes in the power range between −2.00 D and −1.00 D, no statistically significant differences were found. This suggests equivalent visual performance between the two IOLs under photopic conditions, with no significant compromise in CS due to pupil enlargement.

These findings partially contrast with those reported by Pérez et al. [20], who identified statistically significant differences between the ISOPure and MicroPure IOLs at vergences of −1.50 D and −1.00 D with a 3.0 mm pupil, and at −1.50 D and −1.00 D with a 4.5 mm pupil. Specifically, their study reported that the ISOPure lens showed a reduction in CS within the defocus range of −2.00 D to +0.50 D when measured with a 4.5 mm pupil, while the MicroPure lens maintained a more stable response across both pupil sizes. In contrast, the data from the present study do not show a significant drop in CS with increased pupil size in the ISOPure group. This discrepancy may be due to several factors, such as differences in patient characteristics between studies (e.g., age, tear film quality, lighting conditions).

Finally, regarding the overall patient-reported outcomes from the Catquest-9SF questionnaire, the vast majority of patients in both groups expressed satisfaction with their vision after surgery, with no statistically significant differences between them in the global items. However, when analyzing specific items related to daily activities—such as reading subtitles on television, checking prices in stores, or recognizing faces at intermediate distances—the ISOPure group scored better, reinforcing its superiority within the functional range of intermediate vision.

One of the main limitations of the present study is its relatively small sample size. However, this was determined based on robust methodological criteria and is supported by previously published data, which reinforces the validity of the findings. Assessments were conducted at 3 months post surgery, which limits the ability to evaluate the long-term stability and progression of the results. Extending the follow-up period would be valuable for gaining further insight into long-term outcomes.

## 5. Conclusions

Overall, the results of the present study confirm that the ISOPure intraocular lens provides superior visual performance in the intermediate vision range compared to the MicroPure lens, while maintaining distance visual acuity equivalent to that of conventional monofocal lenses, without compromising optical quality. This advantage was evident under both photopic and mesopic conditions. The improvement observed in intermediate vision, together with the good optical quality and the subjective satisfaction reported by patients, positions the ISOPure lens as an effective option for individuals with high intermediate visual demands in their daily lives, such as the use of electronic devices or reading at intermediate distances.

## Figures and Tables

**Table 1:** Demographic and preoperative ocular data of the 28 participants implanted with each IOL.

	ISOPure	MicroPure	*p*-Value
Age (years)	76.8 ± 79.0 (65/90)	75.3 ± 74.0 (64/91)	0.29
Sex (Male/Female) %	50/50	43/57	0.71
UDVA (logMAR)	0.45 ± 0.23 (0.10/0.70)	0.55 ± 0.31 (0.14/1.25)	0.18
CDVA (logMAR)	0.26 ± 0.14 (0.04/0.50)	0.25 ± 0.18 (0.06/0.52)	0.36
LOCSIII	NO/NC 1.0–2.5, C/P <1.0 (35.7%) NO/NC 2.5–3.5, C/P 2.0–4.0 (50%) NO/NC > 4.0, C/P > 4.0 (14.3%)	NO/NC 1.0–2.5, C/P <1.0 (28.6%) NO/NC 2.5–3.5, C/P 2.0–4.0 (42.8%) NO/NC > 4.0, C/P > 4.0 (28.6%)	
IOL Power (D)	22.00 ± 2.27 (18.50/25.50)	21.21 ± 1.77 (17.00/23.50)	0.15
Axial length (mm)	23.31 ± 0.82 (21.67/24.55)	23.62 ± 0.98 (22.13/25.43)	0.18
Corneal astigmatism (D)	−0.47 ± 0.18 (−0.89/−0.22)	−0.54 ± 0.23 (−0.94/−0.12)	0.17
Photopic pupil diameter (mm)	3.39 ± 0.66 (2.49/4.80)	3.46 ± 0.68 (2.34/4.34)	0.38

Values expressed (except sex) as Mean ± SD (maximum/minimum). SD: Standard deviation; UDVA: Uncorrected distance visual acuity; CDVA: Distance-corrected visual acuity; IOL: Intraocular lens; NO/NC: nuclear opalescence/nuclear color; C: Cortical; P: Subcapsular posterior; D: Diopters; mm: Millimeters. Statistical significance: *p* < 0.05.

**Table 2:** Postoperative monocular logMAR visual acuities at different distances under photopic and mesopic luminance conditions in experimental and control groups. Mean ± SD; (range). Only one eye per patient was included.

Monocular Visual Acuity (logMAR Units)						
	Photopic (85 cd/m^2^)			Mesopic (3.5 cd/m^2^)		
	ISOPure	MicroPure	*p*-Value	ISOPure	MicroPure	*p*-Value
UDVA (4 m)	0.02 ± 0.09 (−0.08/0.18)	0.02 ± 0.05 (−0.04/0.14)	0.82	0.16 ± 0.13 (−0.04/0.48)	0.14 ± 0.09 (0.08/0.28)	0.38
CDVA (4 m)	−0.01 ± 0.07(−0.10/0.10)	−0.02 ± 0.06 (−0.16/0.06)	0.63	0.07 ± 0.05 (−0.06/0.14)	0.09 ± 0.08 (−0.04/0.24)	0.19
UIVA (80 cm)	0.17 ± 0.14 (0.00/0.54)	0.25 ± 0.09 (0.10/0.42)	0.05	0.31 ± 0.12 (0.10/0.58)	0.33 ± 0.09 (0.12/0.46)	0.3
DCIVA (80 cm)	0.18 ± 0.06 (0.10/0.34)	0.22 ± 0.06 (0.10/0.36)	0.05	0.36 ± 0.09 (0.20/0.60)	0.33 ± 0.09 (0.14/0.50)	0.25
UIVA (66 cm)	0.25 ± 0.12 (0.05/0.52)	0.30 ± 0.09 (0.12/0.52)	0.02 *	0.37 ± 0.10 (0.20/0.56)	0.45 ± 0.09 (0.30/0.56)	0.02 *
DCIVA (66 cm)	0.20 ± 0.13 (0.00/0.52)	0.25± 0.09 (0.10/0.52)	0.05	0.42 ± 0.11 (0.26/0.66)	0.42 ± 0.10 (0.20/0.58)	0.43

Values expressed as Mean ± SD (maximum/minimum). SD: Standard deviation; UDVA: Uncorrected distance visual acuity; CDVA: Corrected distance visual acuity. UIVA: Uncorrected intermediate visual acuity; DCIVA: Distance-corrected intermediate visual acuity; cm: Centimeters; m: Meters. * Statistically significant difference: *p* < 0.05.

**Table 3:** Postoperative halometry measurements with Halo V1.0 program and simulation software after IOLs implantation in experimental and control groups. Mean ± SD; (range).

		ISOPure	MicroPure	*p*-Value
Halo v1.0	Discrimination index (quadratic)	0.88 ± 0.05 (0.80/0.99)	0.93 ± 0.07 (0.73/1.00)	0.05
Halo and glare software simulator	Halo size	38.00 ± 16.70 (0/38)	41.00 ± 26.36 (2/41)	0.06
	Halo intensity	34.64 ± 24.41 (0/80)	35.5 ± 23.59 (0/77)	0.46
	Glare size	14.71 ± 14.96 (0/45)	25.29 ±18.49 (1/67)	0.06
	Glare intensity	28.71 ± 24.22 (0/66)	31.71 ±22.75 (0/64)	0.6

Values expressed as Mean ± SD (maximum/minimum). SD: Standard deviation Statistically significant difference: *p* < 0.05.

**Table 4:** Optical quality values, including objective scattering index, modulation transfer function, Strehl ratio, and total higher-order aberrations for different pupil diameters, measured 3 months after surgery in experimental and control groups.

	ISOPure	MicroPure	*p*-Value
OSI	1.37 ± 0.77 (0.6/3.6)	1.10 ± 0.31 (0.6/1.70)	0.12
MTF	34.18 ± 10.37 (13.84/51.46)	37.41 ± 7.6 (22.61/46.43)	0.17
Strehl ratio	0.17 ± 0.46 (0.10/0.27)	0.18 ± 0.02 (0.11/0.22)	0.29
HOA (3 mm)	0.15 ± 0.04 (0.06/0.32)	0.10 ± 0.08 (0.06/0.32)	0.04 *
HOA (4 mm)	0.30 ± 0.15 (0.14/0.61)	0.18 ± 0.08 (0.08/0.30)	0.008 *
HOA (5 mm)	0.68 ± 0.22 (0.38/1.07)	0.33 ± 0.16 (0.16/0.69)	<0.001 *

Values expressed as Mean ± SD (maximum/minimum). SD: Standard deviation; mm: Millimeters. * Statistically significant difference: *p* < 0.05.

**Table 5:** Outcomes of the Quality of Vision (QoV) test at 3 months after implantation.

		ISOPure				MicroPure				*p*-Value
		1	2	3	4	1	2	3	4	
Glare	Frequency	71.4	21.4	0	7.1	42.9	57.1	0	0	0.2
	Severity	71.4	14.3	14.3	0	42.9	14.3	42.9	0	0.09
	Bothersomeness	71.4	14.3	14.3	0	42.9	50	0	7.1	0.23
Halo	Frequency	50	28.6	14.3	7.1	42.9	35.7	14.3	7.1	0.78
	Severity	50	42.9	7.1	0	42.9	42.9	14.3	0	0.61
	Bothersomeness	64.3	28.6	7.1	0	42.9	57.1	0	0	0.37
Starbursts	Frequency	78.63	14.3	0	7.1	21.4	64.3	14.3	0	0.008 **
	Severity	78.63	14.3	7.1	0	21.4	50	28.6	0	0.005 **
	Bothersomeness	78.6	21.4	0	0	21.4	64.3	14.3	0	0.002 **
Hazy Vision	Frequency	85.7	14.3	0	0	85.7	7.1	7.1	0	0.94
	Severity	85.7	7.1	7.1	0	85.7	7.1	7.1	0	1
	Bothersomeness	85.7	14.3	0	0	85.7	0	7.1	7.1	0.88
Blurred Vision	Frequency	78.6	21.4	0	0	85.7	14.3	0	0	0.62
	Severity	78.6	21.4	0	0	85.7	7.1	7.1	0	0.7
	Bothersomeness	78.6	21.4	0	0	85.7	7.1	7.1	0	0.7
Distortion	Frequency	100	0	0	0	92.9	7.1	0	0	0.31
	Severity	100	0	0	0	92.9	7.1	0	0	0.31
	Bothersomeness	100	0	0	0	92.9	7.1	0	0	0.31
Double Vision	Frequency	92.9	7.1	0	0	85.7	14.3	0	0	0.54
	Severity	92.9	7.1	0	0	85.7	14.3	0	0	0.54
	Bothersomeness	92.9	7.1	0	0	85.7	14.3	0	0	0.54
Fluctuation	Frequency	64.3	35.7	0	0	71.4	14.3	14.3	0	0.91
	Severity	64.3	35.7	0	0	71.4	14.3	14.3	0	0.91
	Bothersomeness	71.4	28.6	0	0	71.4	14.3	14.3	0	0.81
Focusing Difficulties	Frequency	71.4	28.6	0	0	71.4	28.6	0	0	1
	Severity	71.4	28.6	0	0	71.4	21.4	7.1	0	0.9
	Bothersomeness	85.7	14.3	0	0	71.4	21.4	7.1	0	0.33
Depth Perception	Frequency	100	0	0	0	78.6	21.4	0	0	0.07
	Severity	100	0	0	0	78.6	21.4	0	0	0.07
	Bothersomeness	100	0	0	0	78.6	21.4	0	0	0.08

Values expressed as %. Statistical significance ** *p* < 0.01. 1: Never/not at all; 2: Occasionally/a little; 3: Quite often/quite; 4: Very often/very.

## Data Availability

The data can be requested by email from the authors of this research.
